# Experimental study on deformation characteristics and pore characteristics variation of granite residual soil

**DOI:** 10.1038/s41598-022-16672-8

**Published:** 2022-07-19

**Authors:** Song Yin, Jia’ning Huang, Xinming Li, Linjie Bai, Xianwei Zhang, Chunqing Li

**Affiliations:** 1 School of Civil Engineering and Architecture, Zhongyuan University of Technology, Zhengzhou, 450007 Henan China; 2 State Key Laboratory of Geomechanics and Geotechnical Engineering, Institute of Rock and Soil Mechanics, Chinese Academy of Sciences, Wuhan, 430071 Hubei China; 3 School of Engineering, RMIT University, Melbourne, 3001 Australia

**Keywords:** Geology, Petrology, Civil engineering

## Abstract

This paper investigates the regularity of microstructural evolution of Shenzhen granite residual soil in an attempt to determine its macrostructural deformation characteristics. The pore distribution and microstructure characteristics of granite residual soil are examined experimentally under different consolidation stresses, coupled with mercury injection tests and scanning electron microscopy. It is found that a microstructure exists in the granite residual soil and that the structural characteristics of granite residual soil in Shenzhen. The calculated pre-consolidation stress and structural yield strength of the granite residual soil are similar, indicating a phenomenon that resembles over-consolidation in the granite residual soil. This is fundamentally different from the mechanism of conventional over-consolidation in soil. It is also found that the relationship between the increment of mercury in soil samples and the stress of the mercury under different consolidation stresses is mainly trimodal. The significance of the research presented in this paper lies in the development of a clear understanding of the influence of the microstructure characteristics of granite residual soil on its macrostructural deformation, thereby providing a theoretical basis for engineering applications involving granite residual soil and the analysis of foundation deformation.

## Introduction

Granite residual soil is formed by the weathering of carbonate granites under specific climatic and geological conditions. It is widely distributed in certain regions of China, such as Fujian, Guangdong, Yunnan, and Hunan provinces^1^. In the process of soil weathering, the considerable amounts of leached unstable oxides and significant enrichment of more stable compounds such as iron and aluminum cause the soil to become largely reticulated gravel and sandy clay with brown–red, brown–yellow, and grey–white interphases^2^. Granite residual soil has the residual structure of its parent rock. The ingress of free iron oxides into the soil during the process of weathering can easily form a secondary product^3^, which is then cemented or filled between soil particles to form a specific soil structure, referred to as microstructural soil^4^. The structure of such soil is significantly different from that of conventional structural soil^5–8^. As the residual strength of parent rock is weak, and the free iron oxides are prone to change, the structural stability of microstructural soil is poor and the mechanical properties are complicated and variable. Difficulties in determining the mechanical characteristics^9^ of granite residual soil in engineering practice mean that predictions of its strength and deformation characteristic parameters^10^ have been the focus of considerable research in the field of engineering, particularly in geotechnical mechanics^11^.

The deformation characteristics of soil are closely related to its particle composition, proportion, and distribution. The complexity of the microstructure of granite residual soil means that its deformation characteristics and the evolution regularity governing the microstructure cannot be derived from conventional macro-mechanical tests. Specific studies on the microstructure of such soil and its behavior under external loading must be carried out^12–15^. For soil materials, changes in the pore distribution are an important representation of variations in microstructure and particle arrangement. The deformation characteristics of soil are mainly reflected in the evolution of the microstructure under external loading^16^. To date, there have been a number of studies on the correlation between the pore distribution and deformation characteristics of structural soil. These studies tend to combine deformation testing with microstructural tests, such as the mercury intrusion test (MIP) and scanning electron microscopy (SEM). For example, Griffiths and Joshi^17^ studied the distribution of pore size in different clays at different stages of consolidation. The effect of structural differences on the deformation of clay was analyzed from the perspective of the change in the pore size distribution. Delage and Lefebvre^18^ studied the pore changes during the consolidation of Champlain clay samples using MIP and SEM, and explained the correlation between soil particle adjustment and changes in pore characteristics based on analysis of the pore change rule during clay deformation. Lapierre et al.^19^ developed a mathematical formula relating the pore distribution characteristic parameters and the permeability coefficient of undisturbed soil and remolded soil under different consolidation stresses based on mercury injection tests. They stated that there were no unified mathematical models for the pore characteristic parameters and permeability coefficient of undisturbed soil and remolded soil. Based on SEM and MIP tests, Zhang et al.^20^ analyzed the evolution regularity governing the microstructure morphology of Zhanjiang structural clay in the process of deformation, and found that the microstructure evolved in three distinct stages: structural fine-tuning, structural damage, and structural solidification. Their results have provided technical data for foundation engineering using Zhanjiang clay. Peng et al.^21^ analyzed changes in the pore distribution of soft clay under different consolidation stresses through mercury injection tests, and found that the variation range and regularity governing the pore size distribution and microstructure depend on the stress level in the soil.

A review of the published literature confirmed that the deformation characteristics of structural soil and its microstructure are synchronously linked. However, there has been little research on the evolution of the specific structure of granite residual soil under external loads, and the analysis of the macro-deformation mechanism due to microstructural changes is not sufficient. The physical characteristics, mineral composition, microstructure, and pore characteristics of soil are the basic causal factors for the macrostructural deformation of soil. Due to the specific structural characteristics and mineral composition of granite residual soil^22,23^, the evolution of the microstructure as a result of macro-mechanical properties of the soil in the process of interior failure is different from that of other types of structural soil. Clearly, the influence of the residual structure and cemented oxides of the granite parent rock on the structural characteristics of soil is quite distinct from that of other soils^24^. Therefore, there is a strong need for research on the microstructure and macrostructural deformation characteristics of granite residual soil. This is the purpose of the present paper.

This paper investigates the regularity of microstructural evolution of granite residual soil. One-dimensional compression tests were undertaken, followed by SEM and MIP tests, under different consolidation stresses and with different microstructure and pore distribution characteristics. Both the evolution of the microstructure and the deformation of the macrostructure of the soil were examined under different consolidation stress state changes in pore structure and particle distribution. The correlation between the deformation of the macrostructure of the soil and its microstructural pore distribution is analyzed in this paper. The significance of this research lies in the development of a clear understanding of the influence of the microstructure characteristics of granite residual soil on the deformation of its macrostructure, thereby providing a theoretical basis for engineering applications involving granite residual soil and the analysis of foundation deformation.

## Experiments

### Physical properties of soil samples

**Figure 1:**
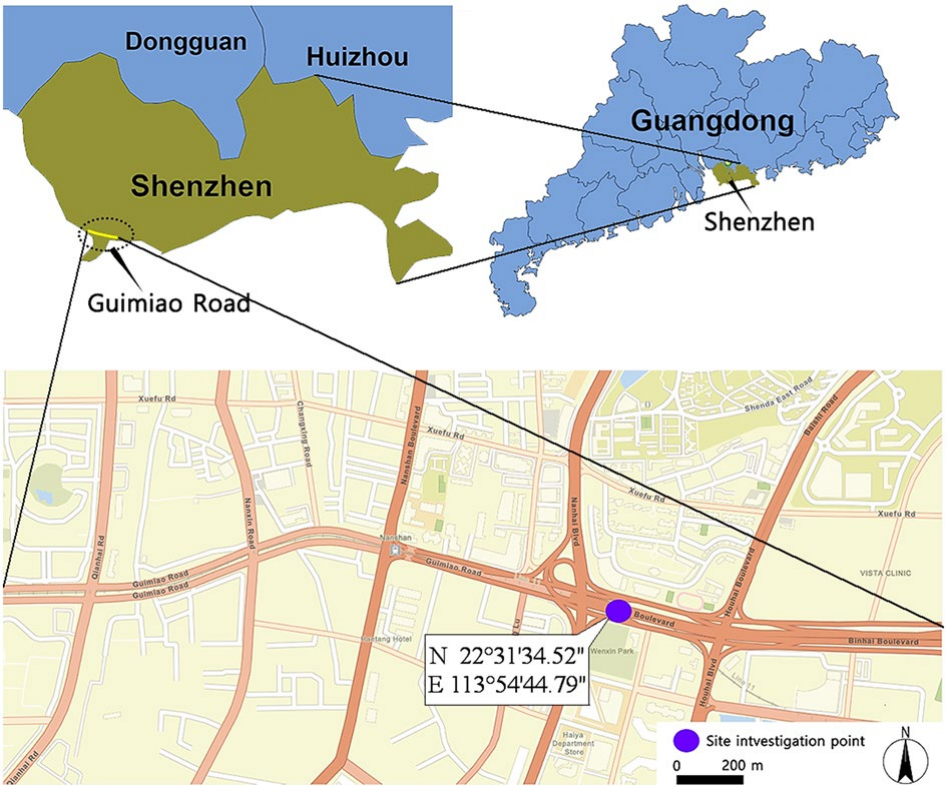
Sampling location in Shenzhen, China. This figure was created with ArcGIS Online (https://www.arcgis.com/apps/mapviewer/index.html) and Pixel Map (http://pixelmap.amcharts.com).

The soil selected in this study was sampled in the vicinity of an engineering site in Nanshan District, Shenzhen City (N22°31′34.52", E113°54′44.79", Fig. 1). Blocks of undisturbed soil (250 mm × 250 mm × 250 mm) for tests were collected at depths of 3.0–4.0 m, the blocks of soil were wrapped in plastic wrap and transported them to the laboratory. Physical indices and the mineral composition are presented in Table 1.

**Table 1:** Average values of physical properties and mineral composition.

Initial saturation (%)	Soil natural density (g/cm^3^)	Natural moisture content (%)	Void ratio	Specific density of solid particles	Liquid limit (%)	Plastic limit (%)	Plasticity index
94.40	1.89	30.54	0.88	2.72	48.80	32.00	16.80

Particle composition (%)				Free oxide content (%)			
Granule (> 2 mm)	Sand (0.075–2 mm)	Silt (0.075–0.005 mm)	Clay (< 0.005 mm)	SiO_2_	Al_2_O_3_	Fe_2_O_3_	Others
7.46	30.44	61.15	0.95	52.56	34.53	9.29	3.62

**Figure 2:**
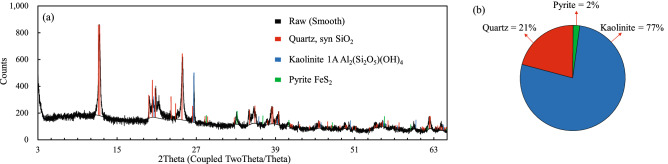
XRD test results. (**a**) XRD characteristics. (**b**) XRD mineral composition ratio.

The selected soil was classified as sandy clayey soil according to the ASTM classification standards^25–28^. X-ray diffraction (XRD) analysis showed that the granite residual soil was mainly composed of the clay mineral kaolinite, with a content of about 77%. Through X-ray fluorescence spectroscopy (XRF) analysis, the soil was found to contain quartz, pyrite, and other non-clay minerals (specific composition given in Table 1 and Fig. 2). Most of the minerals, such as feldspar and biotite, have weathered to kaolinite, indicating a high degree of weathering of Shenzhen granite residual soil after eluviation. The remaining chemical bond strength between loose soil structures is high. The colloid oxides produced by weathering eluviation easily produce cementation between the particles, strengthening the structural connection and forming a microstructure.

### Test methods

#### One-dimensional compression test

Applied a thin layer of Vaseline to the inner wall of the metal ring knife (internal diameter is 61.8 mm, height is 20 mm) used for the test, place the cutting edge down on the sample. Cutter the soil into a cylinder with a soil cutter. The diameter of the soil column is required to be slightly larger than the inner diameter of the ring knife. Then, press down the ring knife vertically, and cut the soil while pressing, until the soil sample is higher than the ring knife, use a wire saw to scrape the soil samples at both ends of the ring knife, and wipe the outer wall of the ring knife. A WG-type single lever consolidation instrument was used to perform one-dimensional compression tests. The saturated samples were subjected to slow compression under stresses of 12.5, 25, 50, 100, 200, 400, 800, and 1600 kPa through a high-pressure consolidation instrument^29^. The displacement of the soil sample at different compressive stress stages was measured by slow consolidation compression (that is, compression for 24 h per stage of compression loading, with changes in gauge reading of not more than 0.005 mm/h considered stable). The samples had a cross-sectional area of 30 cm^2^ and a height of 2 cm.

#### MIP and SEM tests

**Figure 3:**
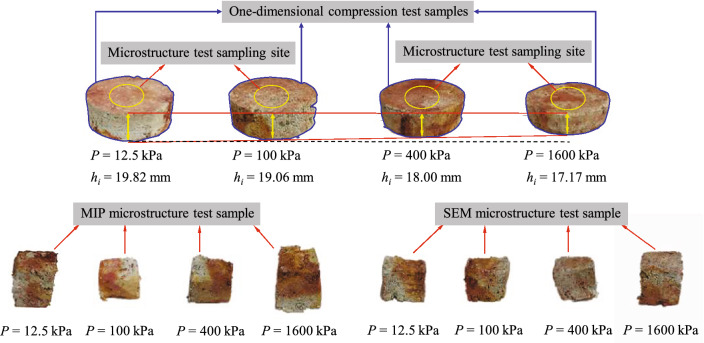
Soil samples for tests.

To analyze the changes in the pore size distribution and microstructure of granite residual soil during the deformation of the macrostructure, samples that had been compressed at 12.5, 100, 400, and 1600 kPa were selected for MIP and SEM tests. The MIP and SEM samples were prepared by freeze-vacuum sublimation drying^30^, as this provides more realistic microstructure information of the soil and avoids the adverse effect of sample drying on the soil structure. A wire saw coated with Vaseline was used to cut samples measuring 10 mm × 15 mm × 10 mm from the specimens used in the one-dimensional compressive tests. A double-sided blade was then used to cut about 1 mm off the perimeter of these samples to obtain the SEM sample. MIP samples measuring 5 mm × 10 mm × 5 mm were cut from the middle section of the prepared SEM samples to ensure a relatively smooth natural structural surface. Figure 3 shows the samples used for the compression, MIP, and SEM tests.

A Micropore Structure Analyzer No. 9310 was used for the MIP tests. This instrument has a pore diameter range of 0.006–300 μm and a stress range of 0.1–60,000 psi (0.6895–413,700 kPa)^20^. As the mercury pressure was gradually increased during the MIP tests, the mercury intrusion volume was monitored. We assumed that the soil pores were cylindrical flow channels, and used the Washburn equation to determine the pore radius associated with each mercury pressure increment. This equation can be expressed as follows:

1 $$ \begin{array}{*{20}c} {R = \frac{2\gamma \cos \theta }{p}} \\ \end{array} $$

where $R$ is the apparent pore radius (nm), $\gamma$ is the surface tension of the mercury (usually 485 × 10^−3^ N/m), $\theta$ is the contact angle between the mercury and the pore surface (140° on average for mercury–air interface in soils), and $p$ is the pressure required to cause the mercury intrusion (MPa). The distribution of the hole radius of the pores was obtained by tracking the amount of non-wetting fluid entering the pore hole as the pressure increased. The cumulative pore volume, pore size distribution, and pore volume percentage were obtained by processing and analyzing the test data.

An SU8100 series Hitachi high-tech ultra-high-resolution field emission scanning electron microscope^24^ was used for the SEM tests. First, the consolidated samples were freeze-dried with liquid nitrogen and sprayed with gold. SEM images with a magnification factor of 2000 × were obtained by using the scanning electron microscope.

### Experimental plan

Six groups of tests were carried out in parallel, among which the first and second groups were one-dimensional consolidation tests with stresses of 12.5–1600 kPa by means of graded loading. The remaining four groups of samples were loaded in the same way as for the one-dimensional consolidation tests. Sample groups 3–6 were taken from the consolidation test samples at loading values of 12.5, 100, 400, and 1600 kPa for the MIP and SEM tests.

### Results and analysis of compression tests

For the consolidation tests, according to the physical parameters of the soil sample, the initial void ratio (*e*_0_) and the void ratio under different consolidation stress (*e*_*i*_) were calculated as follows:

2 $$  e_{0} = \frac{{G_{S} \rho_{w} \left( {1 + \omega } \right)V}}{m}  $$

3 $$ e_{i} = e_{0} - \left( {1 + e_{0} } \right)\frac{\Delta h}{{h_{0} }} $$

where *e*_0_ is the initial void ratio (%), *G*_*S*_ is the specific gravity, *ρ*_w_ is the density of water (T = 4 °C, g/cm^3^), *ω* is the water content (%), *V* is the volume (cm^3^), *m* is the mass (g), *e*_*i*_ is the void ratio under different consolidation stress (%), ∆*h* is the amount of vertical displacement of the specimen under different stresses (mm), and *h*_0_ is the initial height, all of which are with respect to the soil sample (mm).

**Figure 4:**
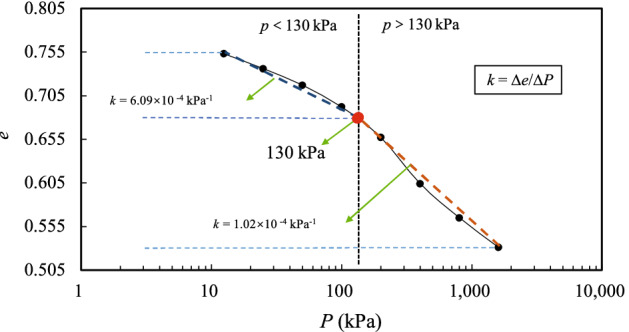
*e*–lg*P* curve.

The change in the void ratio (*e*_*i*_) of the granite residual soil samples with respect to the consolidation stress (*P*) is shown in Fig. 4, in which *k* = Δ*e*/Δ*p*. According to Casagrande’s method, the structural yield strength of soil is about 130 kPa^31^. It can be seen from Fig. 4 that, when the stress reaches 130 kPa, the rate of decrease of the void ratio drops. As the consolidation stress increases, the decrease in the porosity ratio tends to slow. Figure 4 also shows that when the external load on the soil reaches a certain point, the compressibility of the soil decreases. Overall, although the range over which the void ratio of the soil decreases for various stresses varies (as shown by the *e*–lg*P* curve), it is significantly different from that of the conventional structural soil *e–*lg*P* curve^32^. The relative change of the void ratio is small. The results suggest that granite residual soil has a different structure. When the consolidation stress exceeds the structural yield strength of the soil, the structure of the soil is destroyed, and continuous loading causes compaction of the soil. However, compared with conventional structural soil, granite residual soil has a weak structure, and hence belongs to the class of microstructural soils. This finding was confirmed by a recent study on the regularity of microstructural evolution of granite residual soil^33^.

**Figure 5:**
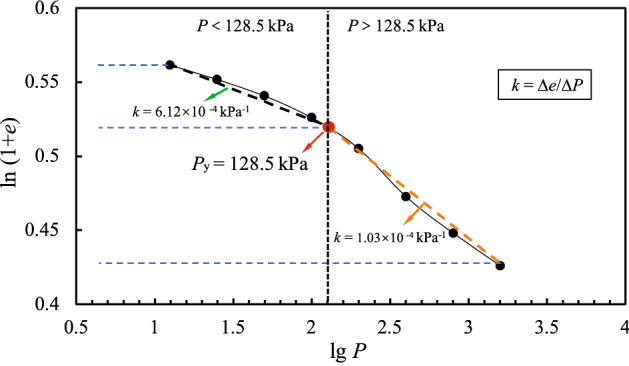
ln(1 + *e*)–lg*P* curve.

Using the double logarithmic coordinate method proposed by Butterfield^34^, the structural yield strength of the granite residual soil was calculated to be 128.5 kPa. The corresponding compressibility coefficient *α*_1–2_ = 0.17 MPa^−1^, indicating that the soil is moderately compressible (Fig. 5, in which *k* = Δ*e*/Δ*p*.).

The two methods gave similar value of the consolidation yield strength, with an average value of approximately 129.3 kPa. As the sampling depth was 3–4 m and the overlying stress was about 80 kPa, the pre-consolidation stress of the soil must be greater than the overlying stress, and the over-consolidation ratio is about 1.6. Because of the specific formation process and surrounding environment of granite residual soil, it is easy to determine that the over-consolidation phenomenon is not caused by the stress history, but is instead a “pseudo-over-consolidation” caused by the residual structural strength after parent rock weathering and the cementation characteristics of free iron oxide. This is fundamentally different from the mechanism of conventional over-consolidation in soil^35^.

## Results and analysis of MIP tests

### In–out mercury curve

**Figure 6:**
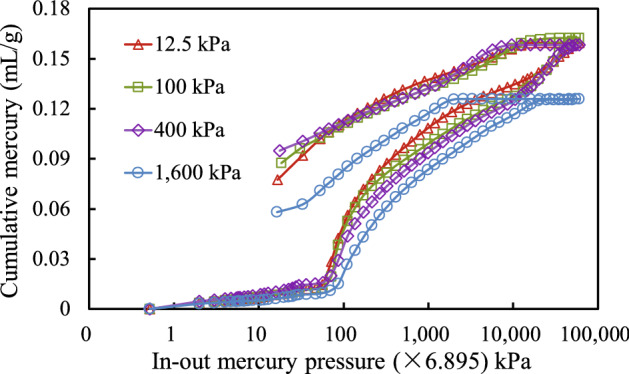
Relation between in–out mercury pressure and cumulative mercury.

Figure 6 shows the relationship between the volume of mercury passed in and out of granite residual soil with respect to the pressure. The cumulative amount of mercury in the sample gradually increased as the mercury pressure increased, indicating that mercury was being compressed into smaller and smaller pores with increasing mercury pressure. As the consolidation stress increased, the cumulative amount of mercury in the samples under the same mercury pressure generally decreased, indicating that the porosity in the samples was gradually decreasing.

The mercury-out curve lies below the mercury-in curve over a certain pressure range. This is mainly because the mercury injection process caused the soil sample to absorb a lot of energy. When mercury was removed, as the pressure decreases, the soil sample matrix stress was released and the sample expanded, and the resulting cracks or void spaces were filled by mercury. Part of the mercury remained permanently in the pores of the soil due to these cracks or void spaces.

**Figure 7:**
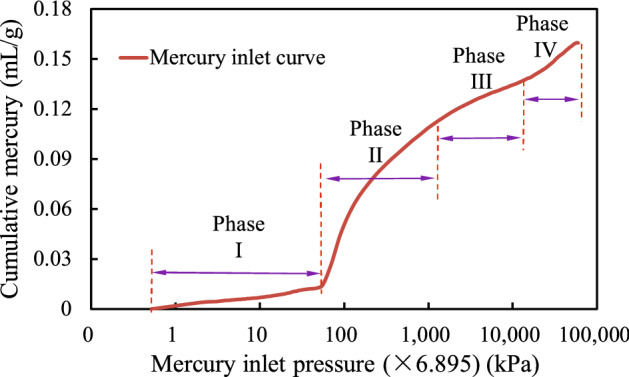
Relation between mercury inlet pressure and cumulative mercury (1 psi = 6.895 kPa).

The relationship between the mercury injection volume and pressure is schematically shown in Fig. 7. The mercury injection process can be generally divided into four stages. In phase I, with low mercury injection pressure, the mercury mainly filled the pore volume between particle interspaces, which are mainly large pores. In phase II, the mercury inflow pressure increased, and some macro pores and medium pores quickly filled with mercury. In phase III, as the mercury injection pressure increased to a certain point, the small and medium pores in the soil sample matrix became filled with mercury due to the high pressure. In phase IV, the mercury injection pressure was very high, and the small pores in the soil were also filled.

Through the multi-stage relationship between the volume of mercury injected into the soil and the pressure, it can be found that the Shenzhen granite residual soil has a large pore size distribution range, and the pore content of each pore size range is obviously different.

**Figure 8:**
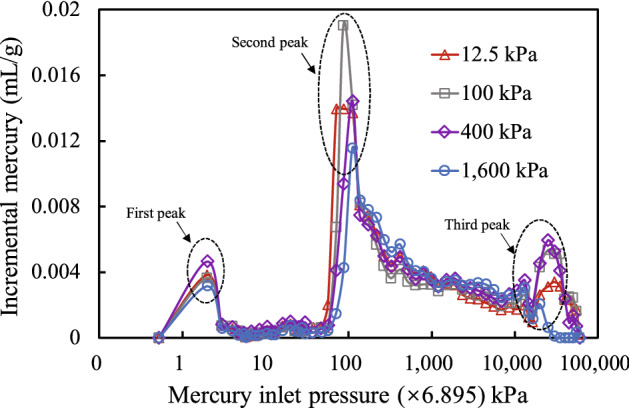
Relation between mercury inlet pressure and incremental mercury.

Figure 8 shows the relationship between the mercury inflow increment and mercury inflow pressure at different consolidation stresses. The distribution of mercury increment with mercury pressure under different consolidation stresses displays triple peaks. As the mercury injection pressure increased, the volume increment increased to a first peak and then rapidly decreased to close to zero. When the mercury injection pressure was increased to 700 psi (4826.5 kPa), the volume increment suddenly increased to a second peak (the maximum peak), and then decreased gradually before increasing to a third peak, and then finally returning to zero when the mercury injection pressure reached 20,000 psi (137,900 kPa). This phenomenon is mainly because the pore structure in granite residual soil has different sizes and shapes. The grain size distribution and mineral elements in the soil are less uniform than those of the parent rock, which has rich iron and manganese nodules, and cementation occurs in the free iron oxide minerals through complex and multifarious combinations. Thus, the diversified contact formation of the complex structure produces an irregular pore size distribution. Under certain external load conditions, the overall pore distribution is relatively unaffected due to the self-adjustment of pore sizes at all levels in the soil.

### Pore distribution of soil

Based on the relationship between mercury injection pressure and volume shown in Fig. 6, the relationship between the pore volume and pore sizes above a certain value and between the pore volume and the distribution of the soil pore content can be obtained. These relationships are illustrated in Figs. 10 and 11, respectively.

**Figure 9:**
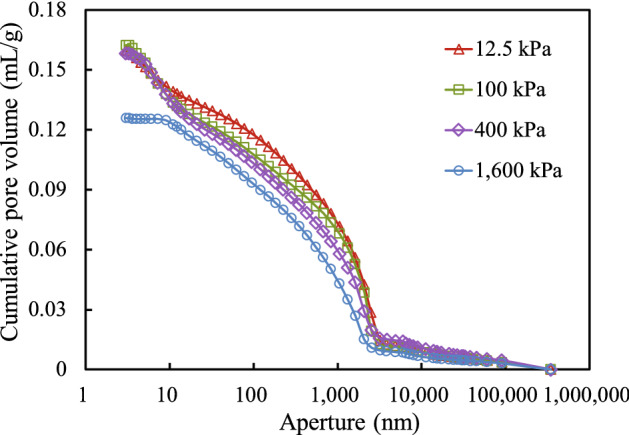
Relationship between aperture and cumulative pore volume.

Figure 9 shows that for consolidation stresses from 12.5 to 400 kPa, the relationship between the cumulative pore volume and pore size remained similar. However, when the consolidation stress was 1600 kPa, the cumulative pore volume for pore sizes of less than 10 nm was significantly lower than that of soil samples under other consolidation stresses. In general, for the same pore size, the cumulative pore volume decreased as the consolidation stress increased, especially in the range of 10–3000 nm, indicating that the total pore volume decreases as the consolidation stress increases in this pore size range.

**Figure 10:**
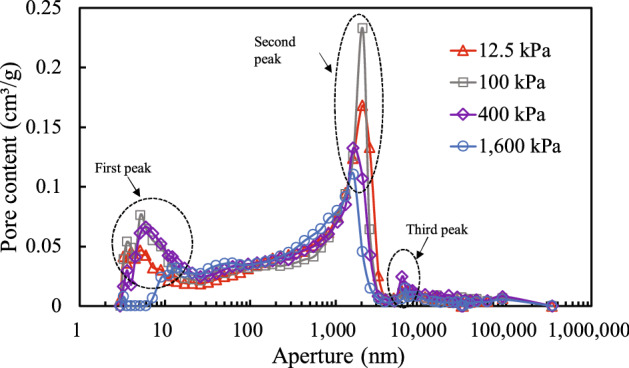
Relationship between aperture and pore content.

Figure 10 shows the pore content distribution of the soil under different consolidation stresses. The pore size distribution of the granite residual soil in Shenzhen mainly ranges from 3 to 400,000 nm, with pore sizes of 2–3000 nm constituting a large proportion. The pore content distribution of soil samples under different consolidation stresses exhibits a three-peak pattern, in which the main peak was for the pore size range from 1000 to 3000 nm, the secondary peak was for the range from 3 to 10 nm, and the small peak was for the range from 9000 to 30,000 nm. For the soil samples studied, the secondary peak corresponded to micro pores, while the main peak corresponded to medium-size pores. The pore size range between the main and secondary peaks corresponded to small pores, and the smallest peak corresponded to large pores. As can be seen from Fig. 10, the differences in the pore size distribution of soil samples with different consolidation stresses are mainly reflected in the different peak values and ranges of the pore size distribution.

### Pore size variation

**Figure 11:**
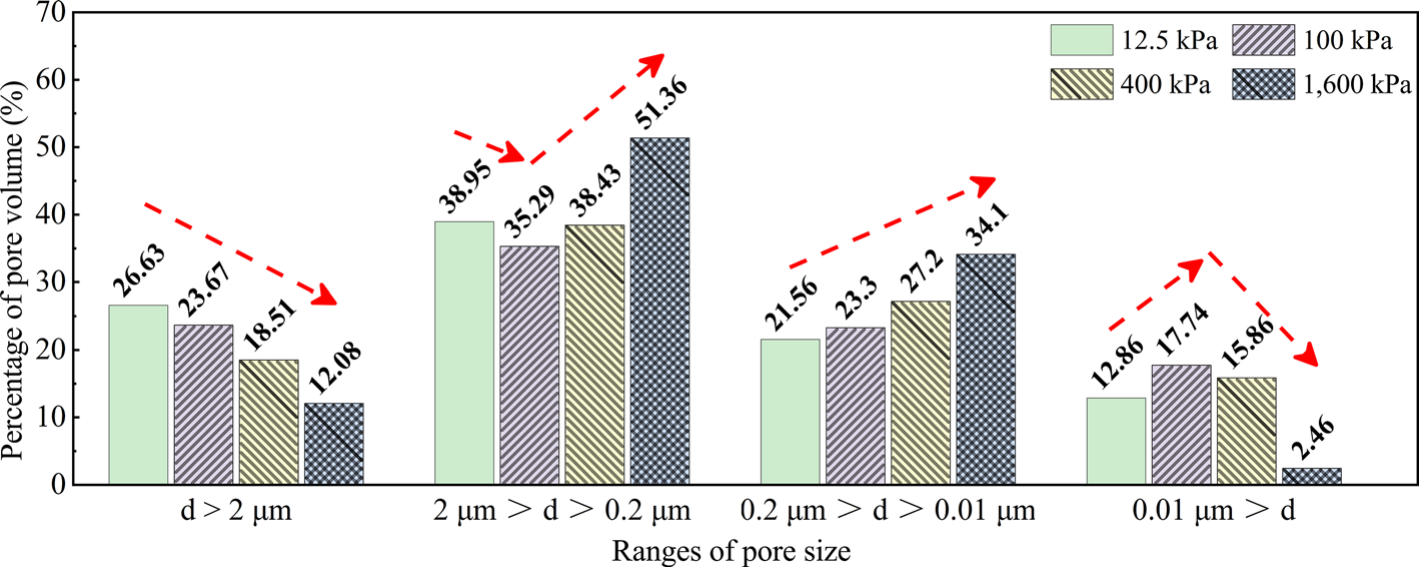
Variation of pore size of soil under different consolidation stresses.

According to the classification standards of the internal pores of soil^36,37^, and based on the shape and variation of the pore size distribution of soil obtained from the mercury injection tests, the pore size ranges of granite residual soil under different consolidation stress were classified in terms of the pore size d as follows: micro, d < 0.01 $\mu \mathrm{m}$; small, 0.01 $\mu \mathrm{m}$ < d < 0.2 $\mu \mathrm{m}$; medium, 0.2 $\mu \mathrm{m}$ < d < 2 $\mu \mathrm{m}$; macro, d > 2 $\mu \mathrm{m}$. Figure 11 shows the variation in the pore size of soil under different consolidation stresses.

As the consolidation stress increased, the volume of the macro pores gradually decreased; the proportion of medium-volume pores decreased at first and then increased; the proportion of small-volume pores increased gradually; and the proportion of micro pores increased slightly at first and then decreased sharply. In general, when the consolidation stress was below the yield strength of the soil, the proportion of large and medium pore volumes decreased, while the proportion of micro and small pore volumes increased. When the consolidation stress exceeded the yield strength of the soil, the volume content of the large and micro pores decreased significantly, while the proportion of medium and small pore volumes increased.

Due to the high degree of weathering of the residual granite soil in Shenzhen, colloid oxides produced by weathering eluviation result in cementation between the particles, strengthening the structural connection and forming a microstructure. Some of the structural characteristics of the original minerals will have been retained. The existence of these microstructures is the reason for the existence of large and medium pores. Before the original structure was destroyed, the external load was mainly borne by the structure of the soil. As the consolidation stress increased, the large and medium pores were compacted into small and micro pores, causing the proportion of large and medium pores to decrease gradually, while the proportion of small and micro pores rose. Once the consolidation stress exceeded the structural yield strength of the soil, a further increase in stress led to a reduction in structural strength. After the macro pores had been destroyed, they became medium and small pores, while the proportion of micro pores basically remained constant. When the stress significantly exceeded the yield strength (1600 kPa), the structure of the soil was seriously damaged, and the bearing capacity of the structure decreased markedly. The macro pores then became medium and small pores, but few reduced to the level of micro pores. Therefore, the proportion of macro pores decreased, while the proportion of medium and small pores increased, and the proportion of micro pores decreased.

The results in Fig. 11 show that the deformation of the macrostructure of granite residual soil under external loading was mainly caused by changes in pore size between soil particles. Due to the unique formation process of this soil, there are obvious differences in the regularity governing the volume change of pores of different sizes.

### Changes in pore specific surface area of soil

**Figure 12:**
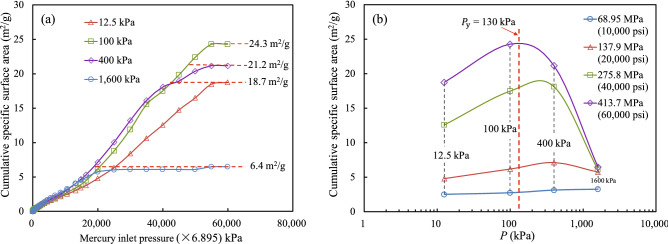
(**a**) Relationship between cumulative specific surface area and mercury inlet pressure. (**b**) Relationship between cumulative specific surface area and consolidation stress.

Figure 12 shows the relationship between the pore specific surface area and the mercury injection pressure for the soil samples under different consolidation stresses, as obtained by collating the mercury injection test data in Fig. 6. For mercury pressures ranging from 0 to 20,000 psi (137,900 kPa), the cumulative specific surface area increased in a consistent and approximately linear manner under different consolidation stresses. Figure 12a shows that when the mercury pressure was greater than 20,000 psi (137,900 kPa), the specific surface area of the soil samples subjected to consolidation stresses of 12.5–400 kPa increased rapidly with an increase in mercury pressure and then became stable. The sample placed under a consolidation stress of 1600 kPa maintained a stable pore specific surface area as the mercury pressure increased. This is mainly because, for consolidation stresses from 12.5 to 400 kPa, the external load on the soil samples was mainly or at least partly borne by the soil’s structural strength, and the structural damage led to large and medium pores becoming small or micro pores. Therefore, as the mercury pressure increased, the small and micro pores gradually filled, and the specific surface area continued to increase until it became stable. However, the soil sample subjected to a consolidation stress of 1600 kPa suffered serious structural damage because it was in a compacted state in which the large pores mainly became medium and small pores, and the medium pores became small pores. However, the volume proportion of micro pores was small, so the specific surface area of pores changed little as the mercury pressure increased.

Figure 12b shows that when the mercury injection pressure was 10,000 psi (68,950 kPa), the specific surface area of the soil samples changed little with respect to the consolidation stress. The injected mercury mainly filled the macro pores, indicating that the change in the soil macro pore volume with respect to the consolidation stress was relatively small. For mercury pressures from 20,000 psi (137,900 kPa) to 60,000 psi (413,700 kPa), the specific surface area of soil increased at first and then decreased as the consolidation stress increased, reaching a peak when the consolidation stress was equal to the yield strength of the soil structure. This further illustrates that there is a certain microstructure in the soil: the external load on the soil structure is mainly borne by its structural strength before yielding, and the compactness of the soil is low. Once the structure has been destroyed, the compressibility of the soil increases, and the total pore volume decreases. The compressive macrostructural deformation of the soil is mainly caused by the change of large pores to medium and small pores.

### Observations and discussion

**Figure 13:**
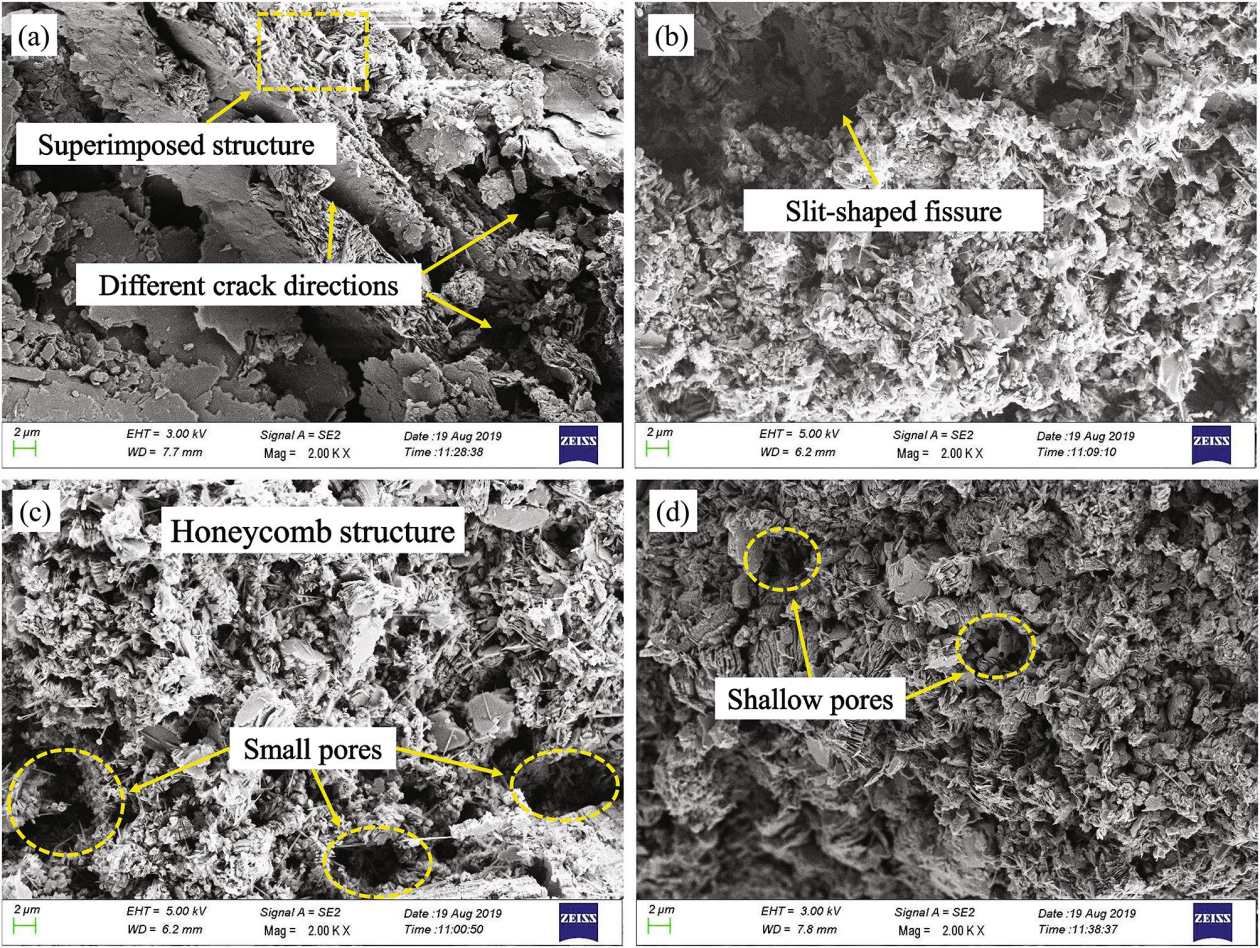
SEM images of soil samples under different consolidation stresses: (**a**) P = 12.5 kPa, (**b**) P = 100 kPa, (**c**) P = 400 kPa, (**d**) P = 1600 kPa.

Figure 13 shows the microstructure of the soil under different consolidation stresses. The microstructure of the granite residual soil (Fig. 13a) is flocculated and honeycombed, with high porosity and many slit pores. The contact between structural units is mainly point-to-point and plane-to-plane, and the soil skeleton is disordered with no obvious orientation. When the consolidation stress increases to 100 kPa (Fig. 13b), there are far fewer slit pores in the soil. The macro pores still exist, and the skeleton structure has remained the same. Under a consolidation stress of 400 kPa (Fig. 13c), the original structure of the soil sample has been largely destroyed and appears to be compacted. The large pores gradually change into small pores with an obvious honeycomb structure. When the consolidation stress reaches 1600 kPa (Fig. 13d), the soil particles have been rearranged in an orderly and enclosed mosaic structure, with a large number of isolated shallow pores and poor connectivity.

In general, the macroscopic performance of the intergranular forces of granite residual soil is represented by *P*_y_. In Fig. 13, when *P* < *P*_y_, an increase in the consolidation stress destroys the connection between particles, large particles and soil aggregates collapse and slip, and more small particles are formed through adjustment and rearrangement. At the same time, these particles move and roll, and the large and medium pores are divided and compressed. This is consistent with our analysis that the proportion of medium and large pore volumes decreases while the proportion of micro and small pore volumes increases (see pore classification criteria in Fig. 11). When *P* > *P*_y_, as the consolidation stress continues to increase, the accumulation of residual soil particles changes the function of intermediate secondary carbonate and free oxides from bonding to filling. The soil skeleton is compressed, and the contacts among structural units are mostly point-to-point. Additionally, there are a large number of isolated, discontinuous, and poorly connected pores. This is consistent with the results of the mercury injection tests, and demonstrates that the soil structure changes from an agglomeration to a honeycomb form, and that the large pores mainly become medium and small pores and the medium pores become small pores.

**Figure 14:**
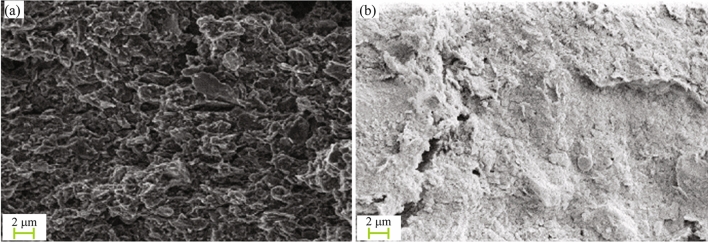
Structural soils. (**a**) Structural clay. (**b**) Structural red clay^2,38^.

Conventional structural soils are formed through long-term consolidated sedimentation, with the soil structure mainly composed of clay cementation, sedimentary load, and deposits formed by various geologic forces with a special layered and honeycomb structure. The structure is relatively intact, and there is strong bonding between particles and a connection between small particles (Fig. 14). However, there are a large number of acicular dispersed minerals and small unknown materials in the granite residual soil of Shenzhen, and these create a unique spatial structure through the contact between particles and the cementation effect of abundant free iron oxides, known as cement pellets. At the same time, the residual structure of the granite parent rock persists in the soil, and certain pores are formed inside the granule aggregates. A specific flocculation structure and honeycomb structure are formed between the structural units of granule aggregates through cementation. The structure formed by overlapping soil particles is complex and the pores are widely distributed, as shown in Fig. 11. Therefore, the structural stability of this type of soil is not as good as that of conventional structural soil, and the structural strength is lower. This type of soil possesses some structural properties, but is relatively weak. Under the action of an external load, the pore adjustment process of the soil is more complicated than that of conventional soil. As the microstructure of the soil determines its macroscopic mechanical behavior, the deformation and pore distribution characteristics of Shenzhen granite residual soil under the action of external loading are different from those of conventional structural soil.

## Conclusion

This paper has described the pore distribution and microstructure of granite residual soil during its macrostructural deformation. The following conclusions can be drawn from the experimental results presented herein.

- There exists a microstructure in the granite residual soil of Shenzhen. The calculated pre-consolidation stress and structural yield strength are similar, indicating a phenomenon that resembles over-consolidation.
- The structural characteristics of the granite residual soil of Shenzhen are different from those of conventional structural soil. The deformation characteristics of the soil are consistent with the response regularity of its microstructure.
- The relationship between the increment of mercury and the stress of mercury in soil samples under different consolidation stresses is trimodal, mainly due to the pore structures of different shapes and sizes.
- The external load on the soil structure before yielding is mainly borne by the structural strength of the soil, such that large pores in the soil are compressed to small pores and medium pores are compressed to micro pores.
- There is a strong correlation between the macro-deformation of soil and the pore distribution of its microstructure.

## Data Availability

Some or all data, models, or code that support the findings of this study are available from the corresponding author upon reasonable request.
